# Towards Biocontained Cell Factories: An Evolutionarily Adapted *Escherichia coli* Strain Produces a New-to-nature Bioactive Lantibiotic Containing Thienopyrrole-Alanine

**DOI:** 10.1038/srep33447

**Published:** 2016-09-16

**Authors:** Anja Kuthning, Patrick Durkin, Stefan Oehm, Michael G. Hoesl, Nediljko Budisa, Roderich D. Süssmuth

**Affiliations:** 1 Technische Universität Berlin, Institut für Chemie, Biologische Chemie, Straße des 17. Juni 124, Berlin, 10623, Germany; 2 Technische Universität Berlin, Institut für Chemie, Biokatalyse, Müller-Breslau-Straße 10, Berlin, 10623, Germany

## Abstract

Genetic code engineering that enables reassignment of genetic codons to non-canonical amino acids (ncAAs) is a powerful strategy for enhancing ribosomally synthesized peptides and proteins with functions not commonly found in Nature. Here we report the expression of a ribosomally synthesized and post-translationally modified peptide (RiPP), the 32-mer lantibiotic lichenicidin with a canonical tryptophan (Trp) residue replaced by the ncAA L-β-(thieno[3,2-*b*]pyrrolyl)alanine ([3,2]Tpa) which does not sustain cell growth in the culture. We have demonstrated that cellular toxicity of [3,2]Tpa for the production of the new-to-nature bioactive congener of lichenicidin in the host *Escherichia coli* can be alleviated by using an evolutionarily adapted host strain MT21 which not only tolerates [3,2]Tpa but also uses it as a proteome-wide synthetic building block. This work underscores the feasibility of the biocontainment concept and establishes a general framework for design and large scale production of RiPPs with evolutionarily adapted host strains.

In the frame of our efforts to generate prototype biocontained strains exhibiting genetic and trophic isolation and expanded biological functions^1^,^2^ we aimed to expand our previous attempts to engineer ribosomally synthesized and post-translational modified peptides (RiPPs) by ribosomally introducing ncAAs into their sequences. Thereby, we are pursuing Xenobiology with the aim to implement various man-made chemical syntheses in living cells. Whereas Synthetic Biology mainly works with naturally occuring building blocks and a canonical chemistry, Xenobiology uses non-natural building blocks and non-canonical chemistries^3^.

Currently, the development of alternative biological systems with radically altered genetic codes implies massive genome engineering^4^. However, approaches aiming at the generation of cell factories as platforms are still immature, as they generally suffer from synthetic lethal mutations, codon reversions and dramatically decreased fitness during the genome assembly process^5^. On the other hand, widely used orthogonal pairs are not as active and accurate as natural aminoacyl-tRNA synthetases with related cognate tRNAs^6^. Our alternative strategy for experimental genetic code evolution towards changes in its biochemistry and to achieve biocontainment relies on the global substitution of canonical amino acids with ncAAs assisted with simple metabolic engineering^7^,^8^. Recently, we described a long-term evolution experiment which led to the reassignment of all 20,899 Trp codons in the genetic code of the bacterium *Escherichia coli*^2^. Cultivation of the *E. coli* strain in defined synthetic media resulted in the generation of the bacterial strain MT21, which is capable of proteome-wide Trp → [3,2]Tpa substitutions in response to all TGG codons in the genome. These evolved bacteria with their new-to-nature amino acid composition are capable of robust growth in the complete absence of the canonical (natural) amino acid Trp (Fig. 1a,b)^2^,^9^. Previously, we and others have applied various methods, aiming to engineer RiPPs by ribosomally introducing ncAAs into their sequences *in vitro* and *in vivo*, exploiting the natural biosynthetic pathways^1^,^10^,^11^,^12^. Nevertheless, supplementation-based incorporation (SPI) only allows for the insertion of isosteric analogues of cAAs, the structural diversity of which is restricted by the promiscuity of the respective tRNA and aminoacyl-tRNA synthetase and limited by the use of auxotrophic strains^13^,^14^,^15^. Expanding the structural complexity of the ncAA regardless of the amino acid to be replaced, can be achieved by stop-codon-suppression (SCS) or reassignment of a sense codon but requires the design of new pairs of orthogonal tRNA and the corresponding aminoacyl-tRNA synthetases^8^,^16^,^17^,^18^,^19^,^20^,^21^,^22^ and genetic modifications such as introduction of the respective codon in the addressed gene and removing of suppressor tRNAs or release factor 1 for improved yields^23^,^24^,^25^,^26^. Herein we report the use of fully adapted *E. coli* MT21 as a platform for production of ncAAs-containing small-molecule-type antibiotic peptides, which undergo massive post-translational modifications, being only recently addressed in the frame of single protein/peptide recombinant production by using standard expression strains^1^,^10^,^11^. The transfer of xenobiological concepts and ideas to peptides with antibiotic properties opens up a new structural space for various compound classes and thus possibly altered or enhanced bioactivities. Peptide natural products, which are ribosomally synthesized and post-translationally modified peptides (RiPPs) comprise of various subgroups, e.g. lanthipeptides^27^,^28^,^29^,^30^, microviridins^31^,^32^, lasso peptides^33^, or linear azole containing peptides^34^,^35^ with various characteristic structural features^36^. We apply the assembly of the otherwise toxic amino acid l-β-(thieno[3,2-*b*]pyrrolyl)alanine ([3,2]Tpa)^37^ (Fig. 1b) to an evolved *E. coli* strain which carries the gene cluster for the heterologous production of the congeneric lantibiotic lichenicidin. Lichenicidin is a two-component lantibiotic originating from *Bacillus licheniformis*^38^. The two peptides, Bliα and Bliβ, are assumed to act synergistically on the cell wall of Gram-positive bacteria in a manner that has been similarly described for other two-component lantibiotics^39^,^40^,^41^,^42^. In this scenario, the α-peptide binds to the peptidoglycan precursor lipid II, and the β-peptide is subsequently recruited to the cell wall to induce pore formation^43^,^44^,^45^. The lichenicidin gene cluster (*lic* cluster, 15 kb) comprises of 14 genes (see Supplementary Fig. S1)^46^, of which only six are essential for heterologous expression of the lichenicidin peptides (Bliα and Bliβ) in *E. coli*^47^. Production of Bliα and Bliβ includes a number of biosynthetic steps (Fig. 1c): subsequent to the ribosomal biosynthesis, an intramolecular crosslinking occurs between dehydrated Ser or Thr and Cys residues to form the diamino diacid lanthionine (Lan) or methyllanthionine (MeLan), respectively. These modifications provide structural stability and rigidity, making lanthipeptides particularly attractive compounds as potential novel antibiotics^48^. The *licA1* and *licA2* structural genes each code for the 72-mer linear precursor peptide of Bliα and Bliβ, respectively. Two sequence-specific modifying enzymes interact with the leader sequence in the corresponding precursor peptide and catalyze the thioether formation in the core region of the respective peptides^46^. A specific membrane transporter protein, carrying a peptidase domain, removes a large portion of the leader sequence prior to the export of the peptide from the cell. An N-terminal hexapeptide remains covalently bound to the modified β-peptide and is not removed until the peptide is translocated outside of the cell, keeping the peptide inactive during the transport. An extracellular protease cleaves off the remaining part of the leader peptide and releases the active peptide (Fig. 1c)^46^.

For the assembly of the Trp-congener [3,2]Tpa (**4**) we chose the β-peptide of lichenicidin, because it naturally carries one Trp in position 9 of the core peptide (see Supplementary Fig. S2). Another advantage is that it is a genetically manageable RiPP system, which can be applied in the heterologous *E. coli* host^49^. According to our approach, by cultivating the evolutionarily adapted strain *E. coli* MT21(DE3) in minimal medium containing a defined set of amino acids with 4*H*-thieno[3,2-*b*]pyrrole (Tp) (**3**) replacing indole (**1**) (Fig. 1b) will increase the selective pressure in favor of translational incorporation of the Trp analogue over Trp into the protein (Fig. 1a). The challenging aspect of our approach is that all of the previously described biosynthetic steps must be able to incorporate [3,2]Tpa globally into all biosynthesized proteins, including those of the post-translational machinery.

## Results

**Figure 1:**
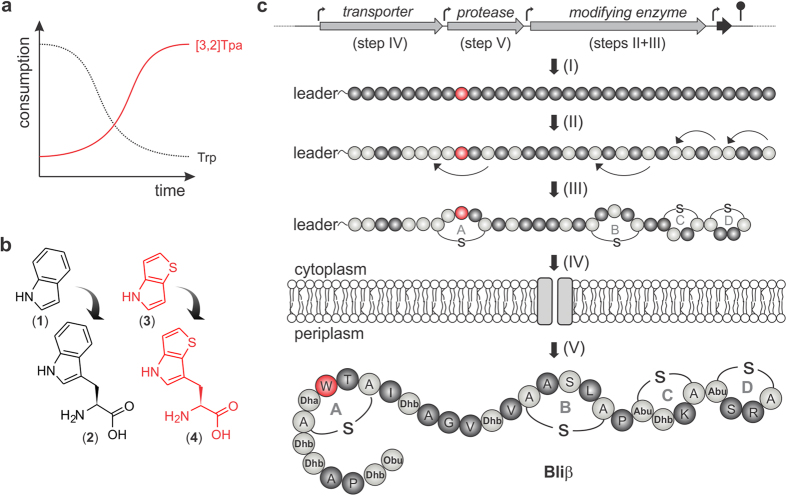
Strategy and prerequisites for the production of congeneric, ribosomally synthesized peptides in emancipated *E. coli* cells. (**a**) Evolutionarily adapted *E. coli* cells are cultivated in defined minimal medium until residual Trp is consumed and cells solely grow on [3,2]Tpa. (**b**) The Trp (**2**) progenitor indole (**1**) is replaced by 4*H*-thieno[3,2-*b*]pyrrole (Tp) (**3**), which in turn is converted into [3,2]Tpa (**4**) by the tryptophan synthetase. (**c**) Scheme of the biosynthesis of Bliβ. The linear precursor peptide is translationally synthesized from the corresponding gene (indicated in black) (I). Dehydrations and thioether bridges are enzymatically installed (II–III) (residues depicted in light grey) and the modified peptide is exported via a specific transporter (IV). Extracellularly, a protease activates the peptide by removal of an N-terminal hexapeptide (V). Dhb, didehydrobutyrine; Dha, didehydroalanine; Obu, 2-oxobutyryl; Abu, aminobutyrate.

**Figure 2:**
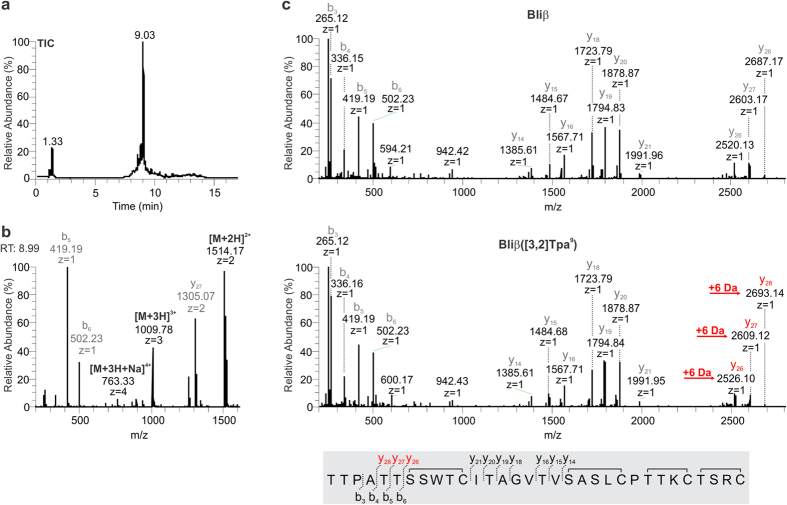
LC-ESI-MS analytics of congeneric Bliβ([3,2]Tpa^9^). (**a**) Total ion chromatogram of Bliβ([3,2]Tpa^9^) extracted with *n*-butanol. (**b**) Mass spectrum of Bliβ([3,2]Tpa^9^) ([M_calc_ + 2H]^2+^ = 1514.17, [M_calc_ + 3H]^3+^ = 1009.78) with annotated fragment masses. (**c**) HR-ESI-MS^2^ analysis of wild type Bliβ ([M + 2H]^2+^ = 1511.18 Da) and congeneric Bli([3,2]Tpa^9^) ([M + 2H]^2+^ = 1514.17 Da). Characteristic mass shifts of 6 Da due to incorporation of [3,2]Tpa as a surrogate for Trp are indicated in red color.

**Figure 3:**
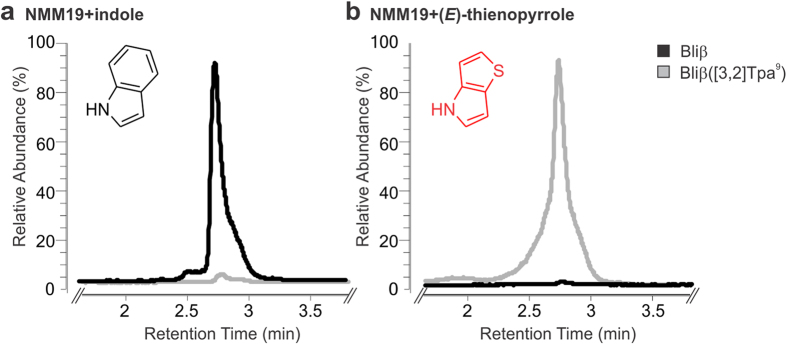
Relative abundance of wild type Bliβ compared to congeneric Bliβ([3,2]Tpa^9^). *E. coli* MT21(DE3) cells were cultivated in NMM19 medium supplemented with (**a**) indole and (**b**) 4*H*-thieno[3,2-*b*]pyrrole (Tp). Peptides were quantified by HPLC-ESI-MS analysis.

**Figure 4:**
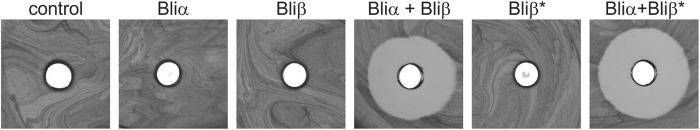
Antimicrobial activity of lichenicidin peptides. Bliα. Bliβ and Bliβ([3,2]Tpa^9^), indicated as Bliβ*, were tested separately (concentration 0.5 μM) and in equimolar (1:1) combinations against the indicator strain *Micrococcus luteus* (DSM-1790). The assay was performed in triplicate.

Cells of strain *E. coli* MT21(DE3) were transformed with the plasmid pRSFDuet-1_*TPM2A2* (see Supplementary Fig. S1), which carries the required genes for Bliβ production in *E. coli*^49^. The resulting strain *E. coli* MT21.1 HPβ was used to express the congeneric Bliβ carrying [3,2]Tpa. The cells were first cultivated in LB medium as a starter culture and subsequently washed and cultivated in minimal medium containing Tp as a precursor for [3,2]Tpa synthesis, until the remaining Trp was consumed (Fig. 1a). Taking the biosynthetic pathway of Bliβ into consideration, we assumed that only the fully processed peptides are exported from the cell and we expected all active peptides to be exclusively located in the culture supernatant. Consequently, the peptides were extracted from the supernatant by addition of *n*-butanol. Indeed, we detected the doubly ([M + 2H]^2+^ = 1514.17), triply ([M + 3H]^3+^ = 1009.78) and quadruply ([M + 3H + Na]^4+^ = 763.33) charged molecular masses of the congeneric peptide by HPLC-MS (Fig. 2a,b). In order to verify the incorporation of [3,2]Tpa into Bliβ, we additionally performed MS/MS experiments, which confirmed the specific mass shift of 6 Da (indole [M_calc_ = 117.06 Da] → 4*H*-thieno[3,2-*b*]pyrrole [M_calc_ = 123.01 Da]) in the A-ring of Bliβ, thus replacing Trp in the peptide (Fig. 2c). In order to assess the specificity, efficiency and the robustness of the expression system we again analyzed the supernatant extracts by means of ESI-MS. When the adapted cells were cultivated in minimal medium with indole as source for Trp synthesis, wild type Bliβ was produced (Fig. 3a). If both, indole and Tp are present in the culture medium, indole is preferably converted into Trp and used for ribosomal synthesis of the peptides (data not shown). When the adapted cells were cultivated in minimal medium supplemented with Tp, the exclusive production of congeneric Bliβ([3,2]Tpa^9^) was observed (Fig. 3b), which exemplifies the robustness of the expression system by not allowing the production of the wild type Bliβ. To assess the bioactivity of this new-to-nature compound, the concentration was determined by mass spectrometric analysis (see Supporting Information). Dried extracts from a cultivation of the same strain in a medium supplemented with indole, instead of Tp, contained wild type Bliβ. We measured the amount of Bliβ proportional to the amount of Bliβ([3,2]Tpa^9^) produced by the strain cultivated in NMM19 + Tp and NMM19 + indole, respectively and observed a 2-fold decrease in production of the congener compared to the wild type (data not shown). In general the peptide yields were much lower than that previously reported^49^, which can be attributed to the limitations of the non-optimal culture medium (NMM19) and genetic modifications necessary for this experimental setup. Considering the differences in the production of Bliβ peptides, we adjusted the amounts of Bliβ and Bliβ([3,2]Tpa^9^) to 0.5 μM and used both in an antimicrobial agar diffusion assay against *Micrococcus luteus* (Fig. 4). As expected, the separate testing of the wild type peptides Bliα and Bliβ did not show any antibacterial effect, while the combination of both peptides resulted in a clear halo indicating antimicrobial bioactivity. Interestingly, the congeneric peptide Bliβ([3,2]Tpa^9^) did not show a decrease in bioactivity, suggesting that the introduction of [3,2]Tpa does not influence the overall structure of the peptide, nor does it negatively affect the interaction with Bliα.

In this study, we firmly prove our working hypothesis, that the application of adapted strains is not only suitable for the expression of a one single protein but also encompasses the possibility for the production of new-to-nature bioactive secondary metabolites, which are synthesized via complex biosynthetic pathways, requiring a relaxed substrate specificity of the PTM machinery for the altered peptide sequence. Moreover, we could demonstrate and confirm the versatile applicability of the complex biosynthesis of lichenicidin, that involves the interaction and catalytic reactions of several proteins, with regard to the exchange of an amino acid with a particular surrogate, beyond techniques aiming at amino acid exchange that have been addressed so far.

## Discussion

Reprogrammed cells or proteins equipped with synthetic structures are currently usually considered as useful tools for academic research or small applications. However, this engineering can even have practical importance when applications such as bioremediation (in open systems) biocatalysts or peptide-based drugs (closed systems) are considered^50^. Furthermore, the incorporation of various ncAAs into the proteome^51^ or in some *E. coli* essential genes^4^,^5^ can be envisioned as a promising biosafety approach: as long as the ncAAs is absent from the medium, no bacterial growth is possible. This is an important prerequisite for biocontainment which is still not fully achieved in our MT21 strain. Namely, it should be noted that 20,899 TGG codons are only trophically reassigned (i.e. the meaning of a codon is redefined throughout the whole translational machinery for the evolved cells only in the defined synthetic medium). That means the supplementation of cells in such a medium with the canonical substrate Trp reverses them to ‘natural’ ones as they still favor the incorporation of the canonical building block. To achieve a nutrient-independent reassignment (i.e. ‘real’ codon reassignment) for the all genome TGG codons in *E. coli* – an experimental strategy for biocontainment needs to be developed and executed.

Nonetheless, for the first time we have provided a solid proof-of-principle for the application of an evolutionarily adapted *E. coli* strain in production of new-to-nature modified lantibiotics. For future bioengineering purposes, our system and its improved versions will doubtlessly provide a manifold of opportunities to design various novel ribosomally synthesized compounds. State-of-the-art modified lanthipeptides are produced (semi-) synthetically^52^,^53^,^54^,^55^, and currently are limited to only a few applications in a healthcare setting^56^,^57^. However, with our methodology we could open up the opportunity to incorporate non-canonical amino acids, enabling us to push forward the *in vivo* diversification of difficult-to-synthesize RiPPs. Recent reports on the development of super-pathogens^58^ emphasize the unabated need for new antibiotics, which can circumvent naturally arising host defense mechanisms^59^,^60^. Hence, the engineering of lantibiotics with chemical structures, rarely occurring in Nature, is a necessary approach to fill the void in developing new antimicrobial compounds^61^.

## Methods

### Cloning

The plasmid pSTB7, carrying the *trpBA* gene originating from *Salmonella typhimurium* which is required for conversion of indole into tryptophan was described earlier^2^. Additionally, we used the vector pRSFDuet-1_*TPM2A2* which carries four genes required for Bliβ production in *E. coli*^49^. For compatibility reasons we exchanged the kanamycin resistance gene of pRSFDuet-1_*TPM2A2* (See Supplementary Fig. S1) for an ampicillin (*amp*) resistance by heterologous recombination applying the arabinose-inducible λ-recombinase system (a kind gift from Dr. Bertolt Gust, Tübingen)^62^. The *ampR* gene was amplified from pET-Duet-1 (Novagen) using the primers AK163 (5′-TTCAAATATGTATCCGCTCATGAGACAATAACCC-3′) and AK164 (5′- TGTGCGCGGAACCCCTATTTGTTTATTTTTCTAAATACATTCAAATATGTATCCGCTCATGAATTAATTCTTACCAATGCTTAATCAGTGAGGCACC-3′).

### Strains

The initial strain used for evolutionary adaption to the non-natural amino acid Tpa was *E. coli* K12 W3110 (CGSC#7679). The generation of thus Trp emancipated strain has been published earlier^2^ and will only be summarized in brief: the genes for the Trp biosynthesis pathway were deleted (∆*trpLEDCBA*) and substituted by *trpBA* on an extrachromosomal plasmid pSTB7. Hence, Trp-synthetase, the gene product of *trpBA*, enables the strain to convert indole into Trp, facilitating to feed the strain either with indole or indole analogues. Adaptation to the indole derivative 4*H*-thieno[3,2-*b*]pyrrole (Tp) finally gave rise to the strain *E. coli* MT21 which continuously could feed on this substrate. As the expression system for lichenicidin requires a T7-polymerase, cells were transformed with a λDE3-lysogenization kit (Novagen, Merck Millipore). The resulting MT21(DE3) cells were transformed with plasmid pRSFDuet-1_*TPM2A2(amp)*.

### Culture Conditions

500 μL of an overnight culture in LB-medium were collected and washed twice in NMM19 medium (7.5 mL 1 M (NH_4_)_2_SO_4_, 68 mL 1 M KH_2_PO_4_, 31 mL 1 M K_2_HPO_4_, 1.7 mL 5 M NaCl, 20 mL 1 M glucose, 1 mL 1 M MgSO_4_, 1 mL Ca^2+^ (1 mg mL^−1^), 1 mL Fe^2+^ (1 mg mL^−1^), 1 mL trace elements, ad 1 L deionised H_2_O, supplemented with 19 canonical amino acids solution, whereupon Trp has been substituted by 4*H*-thieno[3,2-*b*]pyrrole (Tp). Chemical synthesis of Tp has been described earlier^2^. After the second wash the cells were used for inoculation of a 50 mL culture of NMM19 + [3,2]Tp (NMM19 medium supplemented with 0.1 mM of the indole surrogate Tp). The cultures were incubated at 37 °C, 200 rpm until they reached stationary phase. The procedure was repeated for another selection round. From the second 50 mL culture a total of 10 L of main expression culture was inoculated. The main cultures were incubated until optical density was 0.2 at OD_600_. Gene expression was induced by addition of 0.5 mM IPTG (f.c.) and cultures were incubated at 30 °C, 160 rpm for 20 h. For production of wild type lichenicidin the strains *E. coli* HPα and *E. coli* HPβ were cultivated as described earlier^49^.

### Peptide Extraction

Cultures were harvested by centrifugation and supernatant was collected as fully processed congeneric peptides were expected to be exported from the cell. 1/5 volume of *n*Bu was added to the supernatant and incubated shaking. Dried *n*Bu extracts were dissolved in 70% ACN and precipitated in ice-cold acetone for 16 h. Pure Bliα and Bliβ were isolated as described earlier^49^.

### Mass Spectrometric Analysis and Quantification

LC-ESI-MS and LC-ESI-MS^2^ experiments were performed on an ESI-LTQ-Orbitrap (Thermo Scientific). For chromatographic separation a Grom-Sil 120 ODS-5 ST (100 mm × 2 mm, 5 μm) column (GRACE) was used with an Agilent 1260 HPLC system. A gradient starting at 5% solvent B, increasing to 100% solvent B over 10 min, then held at 100% solvent B for 3 min, then over 0.1 min to 5% solvent B followed by 3.9 min isocratic at 5% solvent B was applied with a flow rate of 0.2 mL min-1 (solvent A: H_2_O + 0.1% HFo, solvent B: ACN + 0.1% HFo). MS^2^ spectra were obtained from an IDA Top2 scan using HCD (CE = 35 eV). For quantification LC-ESI-MS/MS using multiple reaction monitoring (MRM) analytics were performed on an ESI-Triple-Quadrupole LC-MS 6460 with a preceding Agilent 1290 Infinity HPLC system (Agilent Technologies). A GRACE Grom-Sil 300 Octyl-6 MB (2 × 50 mm, 3 μm) column was applied for an acetonitrile gradient starting at 5% B, then increasing to 20% B in 0.5 min, then to 70% B in 4 min, and finally to 100% B in 0.2 min, followed by a 1.3 min isocratic hold on 100% B. The flowrate was 0.5 mL min^−1^. For quantitation of lichenicidin peptide yields the [M + 3H]^3+^ adducts of the peptides were used as precursor ions. For MRM the mass transitions for Bliβ m/z 1007.8 → 1302.0, and m/z 1007.8 → 265.1 and for Bliβ([3,2]Tpa^9^) m/z 1009.5 → 1304.5, and m/z 1009.5 → 265.1 were used. Peptide concentrations were compared to a standard curve from purified wildtype Bliβ (see Supplementary Fig. S4).

### Antibacterial Assay

Antibacterial activity was assessed in Mueller Hinton Broth Agar Plates (Difco) against *Micrococcus luteus* DSM-1790 at a final concentration of 0.02 OD_600_. Supernatant extracts from cultures expressing Bliβ or Bliβ([3,2]Tpa^9^) were analyzed by mass spectrometry on an ESI-Triple-Quadrupole with respect to compound concentration and compared to a standard curve. The respective compound was diluted to a final concentration of 0.5 μM and mixed with equal amounts of purified Bliα in 70% ACN and applied to a 5 mm well on the plate. Inhibition zones were determined after 18 h incubation at 30 °C.

## Additional Information

**How to cite this article**: Kuthning, A. *et al*. Towards Biocontained Cell Factories: An Evolutionarily Adapted *Escherichia coli* Strain Produces a New-to-nature Bioactive Lantibiotic Containing Thienopyrrole-Alanine. *Sci. Rep.* **6**, 33447; doi: 10.1038/srep33447 (2016).

## Supplementary Material

Supplementary Information
