# Supplementary material for: Towards Biocontained Cell Factories: An Evolutionarily Adapted Escherichia coli Strain Produces a New-to-nature Bioactive Lantibiotic Containing Thienopyrrole-Alanine
Source: Sci Rep. 2016 Sep 16;6:33447. doi: 10.1038/srep33447 (PMC5025777; doi:10.1038/srep33447)
Supplement: Supplementary Information [file srep33447-s1.pdf]

Supplementary Information for

Towards biocontained cell factories: An Evolutionary Adapted *Escherichia coli* Strain Produces a New-to-nature Bioactive Lantibiotic Containing Thienopyrrole-Alanine

Anja Kuthning[1], Patrick Durkin[2], Stefan Oehm[2], Michael G. Hoesl[2], Nediljko Budisa[2,*], and Roderich D. Süssmuth[1,*]

[1] Technische Universität Berlin, Institut für Chemie, Biologische Chemie, Straße des 17. Juni 124, Berlin, 10623, Germany

[2] Technische Universität Berlin, Institut für Chemie, Biokatalyse, Müller-Breslau-Straße 10, Berlin, 10623, Germany

***** suessmuth@chem.tu-berlin.de and budisa@biocat.tu-berlin.de

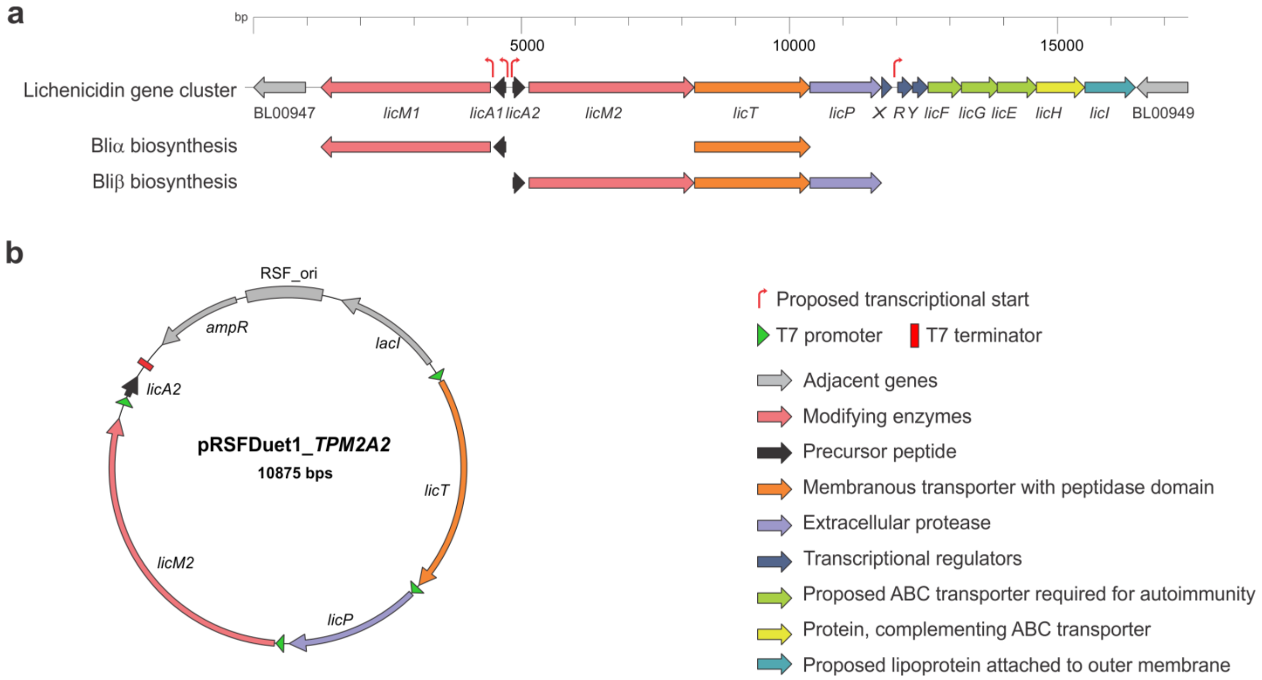

Supplementary figure S1: Genetic background for lichenicidin expression. (**a**) Gene cluster coding for the biosynthesis of lichenicidin as isolated from *Bacillus licheniformis*. Genes required for expression of the single peptides Bliα and Bliβ in *E. coli.* (**b**) Genetic setup for high-level production of Bliβ in *E. coli* as published earlier.

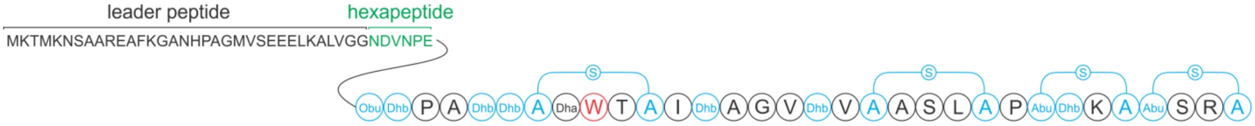

Supplementary figure S2: Amino acid sequence of the full length precursor peptide of Bliβ.

Supplementary table S1: Theoretical and measured nominal masses of Bliβ and Bliβ([3,2]Tpa[9]) detected with high-resolving ESI-Orbitrap-MS.

Compound	Theoretical nominal mass	Measured nominal mass	Mass error (ppm)
Bliβ	3019.3601	3019.3617	0.5
Bliβ[3,2]Tpa	3025.3166	3025.3227	2.03

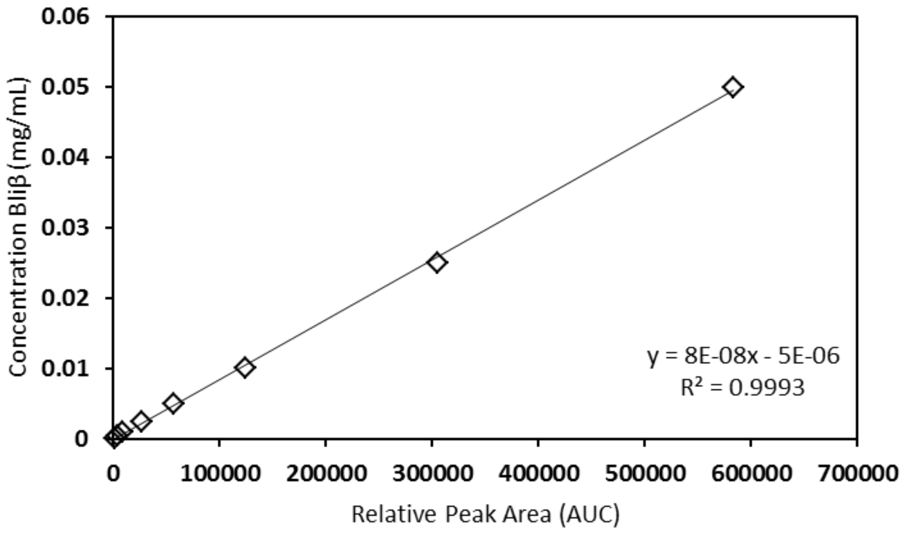

Supplementary figure S4: Bliβ standard curve. Liquid stocks of purified Bliβ peptide were prepared in 70% ACN. Concentrations applied for MS measurements were in the range 0.0001 mg mL^{-1} to 0.5 mg mL^{-1}. The concentration was plotted against the resulting peak area in MRM measurements as stated in the methods section. Line of best fit and coefficient of determination R^2 were calculated as indicated.
